# Genome assembly of a clinical *Fusarium oxysporum* ST33 isolate

**DOI:** 10.1128/mra.00269-26

**Published:** 2026-06-15

**Authors:** Terrance P. Shea, Jeffrey J. Coleman, Christina A. Cuomo

**Affiliations:** 1 Infectious Disease and Microbiome Program, Broad Institute of MIT and Harvard, Cambridge, Massachusetts, USA; 2 Department of Entomology and Plant Pathology, Auburn University 1383, Auburn, Alabama, USA; 3 Molecular Microbiology and Immunology, Brown University 6752, Providence, Rhode Island, USA; University of California Riverside, Riverside, California, USA

**Keywords:** *Fusarium oxysporum*, fungal genome, mycology, human pathogen

## Abstract

The *Fusarium oxysporum* species complex (FOSC) includes agriculturally important pathogens and human-infecting isolates. Here, we report the genome sequence and assembly of a clinical isolate representing ST33. This genome will help with identification and characterization of how some FOSC isolates result in human infections.

## ANNOUNCEMENT

Members of the *Fusarium oxysporum* species complex (FOSC) are notable agricultural pathogens causing wilt diseases on many important crops. Human infections are primarily superficial, resulting in nail or less frequently eye infections; however, in the immunocompromised patient population, life-threatening disseminated infections and fusariosis can occur (1, 2). Based on multi-locus sequence type analysis, the FOSC has been classified into sequence types (ST), where a single worldwide clonal lineage termed ST33 is responsible for a majority of human clinical isolates (3, 4).

A clinical *F. oxysporum* ST33 isolate, FoCl-2, was obtained from a blood culture of a patient with a positive lymph node culture 2 days prior. The isolate was inoculated in 50 mL of potato dextrose broth and grown for 1 week on a rotary shaker at room temperature. Mycelia was harvested by vacuum filtration, and the mycelial mat was lyophilized. High-molecular weight genomic DNA from the isolate was obtained using a cetyltrimethylammonium bromide procedure with a Qiagen Genomic tip 100/G column (5).

A library for Pacific Biosciences sequencing was constructed using the SMRTbell Template Prep Kit 1.0 (Pacific Biosciences) from genomic DNA with no additional shearing or size selection following the manufacturer’s methods. This sample was sequenced on a Pacbio RS to generate a total of 1,033,635 reads with P4 chemistry and 89,902 reads with P5 chemistry; the N50 length of all reads was 9.1 Kb, and the sequence read depth was 123×. All reads were assembled with HGAP 2.0 (6) (full settings provided as an xml on FigShare [https://doi.org/10.6084/m9.figshare.32129800]). The resulting assembly consists of 263 contigs, with an N50 length of 4.1 Mb and a total assembly size of 54.7 Mb. The assembly was aligned to that of *F. oxysporum* f. sp. *lycopersici* 4287 (GCA_000149955.2), a tomato wilt isolate (7), with nucmer (mummer 3.23 [8]) with –nosimplify –maxmatch, and the percentage of reference bases covered in the alignment was calculated. We identified 15 contigs corresponding to the core, shared chromosomes, with the remaining 248 contigs representing regions of lineage-specific chromosomes. Comparison with the 4,494 conserved gene set close to this species (hypocreales_odb10) with BUSCO v6.0.0 (9) found 99.7% complete genes, suggesting this assembly is a highly complete representation of conserved gene content.

## Data Availability

The sequence and assembly reported here are available in GenBank under BioProject PRJNA1112883. Raw sequence reads have been deposited in the NCBI Sequence Read Archive for the PacBio data under SRR29069803 and SRR29069802. The assembly is deposited under accession JBDQYN000000000.
